# Incidence of transfusion‐related acute lung injury temporally associated with solvent/detergent plasma use in the ICU: A retrospective before and after implementation study

**DOI:** 10.1111/trf.17049

**Published:** 2022-08-02

**Authors:** Robert B. Klanderman, Nielsvan van Mourik, Dorus Eggermont, Anna‐Linda Peters, Pieter R. Tuinman, Rob Bosman, Henrik Endeman, Olaf L. Cremer, Sesmu M. Arbous, Alexander P. J. Vlaar

**Affiliations:** ^1^ Department of Intensive Care Amsterdam University Medical Centers – AMC Amsterdam The Netherlands; ^2^ Laboratory of Experimental Intensive Care and Anesthesiology Amsterdam University Medical Centers – AMC Amsterdam The Netherlands; ^3^ Department of Anesthesiology Amsterdam University Medical Centers – AMC Amsterdam The Netherlands; ^4^ Department of Anesthesiology University Medical Center Utrecht Utrecht The Netherlands; ^5^ Department of Intensive Care Amsterdam University Medical Centers – VUmc Amsterdam The Netherlands; ^6^ Department of Intensive Care Onze Lieve Vrouwe Gasthuis – Locatie Oost Amsterdam The Netherlands; ^7^ Department of Intensive Care University Medical Center Utrecht Utrecht The Netherlands; ^8^ Department of Intensive Care Leiden University Medical Center Leiden The Netherlands

**Keywords:** critically ill, FFP, plasma, pulmonary edema, TRALI

## Abstract

**Background:**

Transfusion‐related acute lung injury (TRALI) is a severe complication of plasma transfusion, though the use of solvent/detergent pooled plasma (SDP) has nearly eliminated reported TRALI cases. The goal of this study was to investigate the incidence of TRALI in intensive care units (ICU) following the replacement of quarantined fresh frozen plasma (qFFP) by SDP.

**Study design and methods:**

A retrospective multicenter observational before–after cohort study was performed during two 6‐month periods, before (April–October 2014) and after the introduction of SDP (April–October 2015), accounting for a washout period. A full chart review was performed for patients who received ≥1 plasma units and developed hypoxemia within 24 h.

**Results:**

During the study period, 8944 patients were admitted to the ICU. Exactly 1171 quarantine fresh frozen plasma (qFFP) units were transfused in 376 patients, and respectively, 2008 SDP units to 396 patients after implementation. Ten TRALI cases occurred during the qFFP and nine cases occurred during the SDP period, in which plasma was transfused. The incidence was 0.85% (CI95%: 0.33%–1.4%) per unit qFFP and 0.45% (CI95%: 0.21%–0.79%, *p* = 0.221) per SDP unit. One instance of TRALI occurred after a single SDP unit. Mortality was 70% for patients developing TRALI in the ICU compared with 22% in patients receiving at least one plasma transfusion.

**Conclusion:**

Implementation of SDP lowered the incidence of TRALI in which plasma products were implicated, though not significantly. Clinically diagnosed TRALI can still occur following SDP transfusion. Developing TRALI in the ICU was associated with high mortality rates, therefore, clinicians should remain vigilant.

**Abbreviations**
CABG: Coronary artery bypass graft
COPD: Chronic obstructive pulmonary disease
CVA: Cerebrovascular accident
FiO_2_
: Fraction of inspired oxygen
GI: Gastrointestinal
HLA: Human leukocyte antigen
HNA: Human neutrophil antigen
ICU: Intensive care unit
LOS: Length of stay
P/F‐ratio: PaO_2_/FiO_2_‐ratio
PaO_2_
: Partial pressure of oxygen
PLT: Platelets
qFFP: Quarantined fresh frozen plasma
RBC: Red blood cells
SAPS: Simplified acute physiology score
SDP: Solvent detergent treated plasma
TRALI: Transfusion‐related acute lung injury

## 1 INTRODUCTION

Transfusion‐related acute lung injury (TRALI) is a severe adverse transfusion reaction, defined as the acute onset of pulmonary permeability edema and respiratory compromise within 6 h after transfusion. TRALI develops following a “two‐hit” event, threshold model.^1^, ^2^ Neutrophils in the lungs are primed by a first hit, for example, cardiac surgery, sepsis, or trauma. In TRALI, these cells are subsequently activated (second hit) by antibodies stored specifically in plasma containing blood products. TRALI develops if the sum of the first‐ and second hit is large enough to pass the threshold. Therefore, depending on the patient's predisposition (severity of the first hit), the second hit can be at a lower antibody titer. Patients in the intensive care unit (ICU) are especially at risk of TRALI, as their underlying condition often primes neutrophils, and up to 40% receive at least one blood transfusion during their ICU stay.^3^

Over the past decades, mitigation strategies have lowered the incidence of TRALI. After the introduction of quarantined, single‐donor, male‐only, fresh frozen plasma (qFFP), the incidence decreased by more than 50%,^4^, ^5^ to approximately 5.5% of patients transfused in the ICU.^6^, ^7^ The most recent mitigation step has been the implementation of solvent/detergent treated pooled plasma (SDP).^8^, ^9^, ^10^ SDP is produced by pooling plasma from hundreds of donors, diluting harmful antibodies below detectable levels.^11^ It is further postulated that harmful antibodies may also be neutralized by soluble antigens during pooling. Nevertheless, undetectable antibody titers are not evidence of absent antibodies.

Since SDP replaced quarantine fresh frozen plasma (qFFP) in 2014 in the Netherlands, the incidence of TRALI has dramatically fallen. Until 2020, there have been no cases reported of TRALI due to SDP, which is now widely seen as the safer alternative concerning TRALI.^9^ One study even heralded SDP as having “abolished” TRALI from plasma transfusions.^12^ The Dutch national hemovigilance network recently reported a case of TRALI, though transfusion‐associated circulatory overload could not be ruled out.^13^ We hypothesize that the incidence of TRALI after plasma transfusion has decreased since implementation; however, TRALI can still occur after SDP considering the threshold model. A retrospective before and after implementation study was performed to investigate whether the introduction of SDP has reduced the incidence of TRALI in ICU patients compared with qFFP.

## 2 MATERIALS AND METHODS

This study was approved by the medical ethics committee (Amsterdam University Medical Centers—location AMC—*Amsterdam, The Netherlands*), and informed consent was waived due to the retrospective nature of the study. We performed a multicenter retrospective observational before and after implementation cohort study employing an active retrospective surveillance strategy to identify episodes of TRALI following plasma transfusions. Transfusion episodes of all patients, either already admitted to the ICU or newly admitted, were reviewed during two 6‐month periods: (1) between April 1 and October 1, 2014; and (2) between April 1 and October 1, 2015. Excluded were patients readmitted to the ICU and <18 years of age. The 2014 inclusion window was prior to the implementation of SDP, and all plasma transfusions were units of qFFP. In November 2014, all blood banks nationwide switched to SDP; the second inclusion period was chosen after a 6‐month washout phase.

### 2.1 Plasma products

Male‐only, qFFP (310 ml) was the standard plasma product available nationwide (Q‐Plasma, Sanquin Blood Bank—*The Netherlands*). It is produced from the apheresis plasma of a single, non‐paid, volunteer donor. Implementation of SDP has advantages, including an improved safety profile with regards to pathogen reduction as well as limiting the quantity of harmful antibodies per unit of plasma. The Netherlands only uses Omniplasma® (200 ml) (Octapharma—Switzerland), which follows the same production process as Octaplas® (Octapharma—Switzerland). However, the plasma is from all Dutch, non‐paid, volunteer donors. They are produced from the pooling of 300–500 apheresis plasma donations. Keeping in mind the threshold model for TRALI, rather than exposing one patient to the entire volume of a plasma unit that may have harmful antibodies, pooling of plasma limits the total quantity of antibodies per unit, preventing patient exposure to high antibody titers.

### 2.2 Case selection

Selection of TRALI patients was through a multitiered approach: (1) potential TRALI cases were identified based on electronically recorded patient data; (2) a full chart review was performed for these patients. Data were collected from all patients with evidence of respiratory decline, which was temporally associated with a plasma transfusion (<6 h); (3) a panel with transfusion experts made the final determination of whether cases fulfilled TRALI criteria (Table 1) and assigned imputability (eTable 1).

**TABLE 1:** Revised 2019 consensus redefinition for transfusion‐related acute lung injury

**TRALI type I**
Acute onset
Hypoxemia (P/F‐ratio ≤ 300 or SpO_2_ < 90% on room air)
Clear evidence of bilateral pulmonary edema on imaging (CXR, Chest‐CT, ultrasound)
No evidence of LAH or, if LAH is present, it is judged to not be the main contributor to the hypoxemia
b. Onset during or within 6 h of transfusion
c. No temporal relationship to an alternative risk factor for ARDS
**TRALI type II**
Findings as described in categories *a* and *b* of TRALI Type I, and
Stable respiratory status in the 12 h before transfusion

*Note*: Adapted from Vlaar, Transfusion (2019).^14^

Abbreviations: ARDS, acute respiratory distress syndrome; CXR, chest x‐ray; LAH: left‐atrial hypertension; P/F‐ratio, PaO_2_/FiO_2_‐ratio.

During step 1, potential TRALI patients were retrospectively identified based on electronic patient data. They (1) received at least one plasma transfusion and (2) had a pressure of oxygen (PaO2) and fraction of inspired oxygen (FiO2) (P/F)‐ratio <300 within 24 h prior to or post‐transfusion of the plasma unit. This wide time interval prevented the exclusion of cases in which the electronic times and dates of transfusion time, mechanical ventilation, and blood gas results were not well synchronized. Patients excluded as potential cases were those extubated within 9 h of the plasma transfusion or discharged from the ICU within 48 h of admission and were therefore unlikely to have TRALI, unless they died. Electronic patient data were supplied from participating centers. All Dutch ICUs participate in the collating and reporting of anonymized patient data to the NICE foundation for monitoring and optimizing quality of ICU care nationally (*Stichting NICE*—Amsterdam, the Netherlands), from which patient characteristics, as well as admission diagnosis, APACHE‐II and SAPS scores, type of admission (medical/planned surgical/emergency surgical), referring specialty, major comorbidities including heart, lung or liver disease, and malignancies were derived. Minute‐to‐minute ventilator data were collected, including ventilation mode, as well as FiO_2_ and PaO_2_ values from arterial blood gas analyses of all patients in ICU during the inclusion periods. In case no FiO_2_ was available, it was assumed to be 21%, and PaO_2_/FiO_2_‐ratios (P/F‐ratio) were calculated accordingly. An overview of all transfused blood products (eTable 2) and the time they were administered were queried from the electronic records.

A full chart review was performed on all electronically identified patients (step 2). Two independent researchers reviewed each patient's (R.K, A.P, D.E) charts, selecting only the patients that showed new onset or respiratory worsening temporally associated (<6 h) after a plasma transfusion. Only for these selected patients were additional data collected, thereby excluding patients who showed respiratory compromise either >6 h after transfusion or prior to transfusion. Respiratory worsening was defined as increased oxygen requirements, intubation, decreased P/F‐ratios compared with pre‐transfusion, or worsening ventilatory parameters (including a decrease in compliance, increase in FiO_2_, positive‐end expiratory pressure or driving pressure). Data reviewed and collected included: doctor and nurse notes, respiratory parameters, oxygen requirements, hemodynamic variables (e.g., heart rate, blood pressure and central venous pressure, fluid balance), medication where relevant, start and end times of transfusion, other units transfused and laboratory data including complete blood counts and blood gas results. Additionally, chest X‐rays and chest computed tomographies (CTs), along with the radiology report and echocardiography results where available, were collected.

A panel of transfusion experts and researchers (Niels van Mourik, Anna‐Linda Peters, and Alexander P.J. Vlaar) reviewed the cases and made the final determination by consensus (step 3) on whether patients had developed TRALI according to the 2019 revised TRALI definition (Table 1).^14^ The panel was blinded to the date of the transfusion as well as the type of plasma product transfused. Patients already mechanically ventilated were required to have stable ventilatory parameters for at least 12 h prior to transfusion. The panel excluded patients on extracorporeal life support, since no reliable P/F‐ratios could be determined, and patients who died before any further investigations could be performed (e.g., P/F‐ratio determination or a chest X‐ray performed) combined with an alternate clinical explanation. Imputability for each case of TRALI was scored as either definite, probable, or possible, following the international society for blood transfusion 2011 definitions (eTable 1).^15^ Cases with imputability scored as doubtful were not included.

### 2.3 Outcomes

The primary outcome of our study was the incidence of TRALI after implementation of SDP compared with pre‐implementation. The secondary outcomes included the incidence of TRALI corrected for the difference in volume of the plasma products transfused, risk factors for TRALI, the number of cases that received plasma only, and the imputability of TRALI for these cases. Patient outcomes including mortality, hospital, and ICU length of stay (LOS) were compared between TRALI patients, transfused patients (any transfusion), and plasma transfused patients (receiving at least one unit of plasma, concomitantly with other products or alone).

### 2.4 Sample‐size calculation

Prior implementation of qFFP led to an approximate risk reduction of up to 66%,^4^, ^5^, ^16^ with an incidence thereafter reported in the ICU of 2.4%–8.2%.^7^, ^17^, ^18^ Using a weighted mean incidence, approximately 5.5% of the transfused patients developed TRALI in the ICU due to qFFP. Considering only one case of TRALI due to SDP has been reported to date, we assumed implementation of SDP dramatically reduced the risk of TRALI. Using a chi‐square test with an alpha of 0.05 and 80% power, at least 302 patients transfused per group are required to show a 75% relative risk reduction for the incidence of TRALI. Inclusion of centers continued until the number of patients receiving plasma transfusions was fulfilled.

### 2.5 Statistical analysis

Data were inspected for normality. Binary outcomes were compared using a chi‐square test. To compare continuous data from patients between the qFFP and SDP period, we used a Mann–Whitney U test or unpaired Student t‐test wherever appropriate. A corrected SDP unit count was calculated to correct for a difference in volume between qFFP (310 ml/unit) and SDP (200 ml/unit). The number of SDP units was corrected using the following formula: Corrected SDP units = SDP units transfused (*n*) * (volume SDP unit (ml)/volume qFFP unit (ml)). The statistical analysis was performed in R‐statistics (version 3.3.2) using the R‐studio package. Statistical significance was considered at p < 0.05.

## 3 RESULTS

A total of 8944 patients were included, from a total of five Dutch ICUs, of which four were tertiary academic centers and one was a secondary teaching hospital. A total of 2068 patients received 12,804 blood products. Baseline characteristics and transfusion data of all ICU patients before and after the implementation of SDP are shown in Table 2. During the SDP period, the number of plasma products transfused was significantly higher. This remained significantly higher after correction of plasma units for volume (*p* = 0.013). Post‐hoc analysis showed that this was the result of outliers; the upper range of qFFP transfused in a single patient was 29 units, while for SDP, this was 564 units as part of plasmapheresis. When excluding the four patients who received the highest number of plasma transfusions in the SDP group (total: 741 units, range: 35–564), the number of plasma units transfused was not significantly different between groups (*p* = 0.052); all patients were included in subsequent analyses.

**TABLE 2:** Baseline characteristics

Characteristics	Period		*p*‐value
	qFFP	SDP	
ICU admissions, n	4563	4381	
Age, years	65 (54 to 74)	65 (54 to 73)	0.655
Male, *n* (%)	2896 (63.5%)	2855 (65.2%)	0.085
BMI	26.4 ± 5.0	26.5 ± 5.1	0.184
APACHE‐II Score	15 (12 to 21)	15 (12 to 21)	0.396
SAPS	33 (25 to 45)	33 (26 to 46)	0.081
Products transfused, n	5985	6819	<0.001
RBC units	3694	3765	0.160
PLTs units	1023	1141	0.011
Plasma products	1171	2008	< 0.001
qFFP	1171	0	“
SDP	0	2008	“
Plasma units (vol. corrected)^a^	1171	1294	0.013
Patients transfused, *n* (%)	1068 (23.4)	1000 (22.8)	0.516
Units per patient transfused	3 (1 to 6)	3 (1 to 6)	0.269
RBC units	2 (1 to 4)	2 (1 to 5)	0.742
PLT units	1 (1 to 2)	1 (1 to 2)	0.057
Plasma units	2 (1 to 4)	2 (1 to 4)	0.255
Plasma volume (L/patient)^†^	1.2 (0.6 to 3.1)	1.2 (0.6 to 5.0)	< 0.001
Type of admission, *n* (%)			< 0.001
Medical	1666 (36.5%)	1664 (38.0%)	
Emergency surgery	762 (16.7%)	832 (19.0%)	
Planned surgery	2130 (46.7%)	1885 (43.0%)	
Comorbidities, *n* (%)			
Chronic renal disease	279 (6.1)	244 (5.6)	0.272
COPD	359 (7.9%)	355 (8.1%)	0.698
Hematological malignancy	104 (2.3%)	109 (2.5%)	0.521
Immunological insufficiency	316 (6.9%)	358 (8.2%)	0.032
Diabetes	749 (16.4%)	720 (16.4%)	1.000
History of heart failure	356 (7.8%)	273 (6.2%)	0.006
Cirrhosis	83 (1.8%)	57 (1.3%)	0.044
Risk factors, *n* (%)			
Direct			
Pneumonia	133 (2.9%)	129 (2.9%)	0.954
Aspiration	35 (0.8%)	27 (0.6%)	0.461
Inhalation: smoke/drowning	7 (0.2%)	3 (0.1%)	0.352
Indirect			
Sepsis	187 (4.1%)	190 (4.3%)	0.596
Trauma	182 (4.0%)	176 (4.0%)	0.951
Pancreatitis	8 (0.2%)	11 (0.3%)	0.496
Drug overdose	87 (1.9%)	97 (2.2%)	0.325
Other			
Cardiac surgery	1702 (37.3%)	1621 (37.0%)	0.767
CVA	195 (4.3%)	187 (4.3%)	1.000
Cardiac arrest	216 (4.7%)	212 (4.8%)	0.842
Hospital LOS (days)	9 (5 to 18)	10 (6 to 26)	0.000
ICU LOS (days)	2 (1 to 3)	2 (1 to 3)	0.026
Died in ICU, *n* (%)	437 (9.6%)	421 (9.6%)	0.972

*Note*: Data presented as mean ± SD or median (IQR).

^a^ Calculated plasma volume transfused per patient.

^**†**^ The number of SDP units transfused when correcting for volume (qFFP 310 mL vs. SDP 200 mL).

Abbreviations: ALI, acute lung injury; COPD, chronic obstructive pulmonary disease; CVA, cerebrovascular accident; LOS, length of stay; PLT, platelet transfusion; qFFP, quarantine single unit fresh frozen plasma; RBC, red blood cells; SAPS, simplified acute physiology score; SDP, solvent/detergent treated pooled plasma.

**FIGURE 1:**
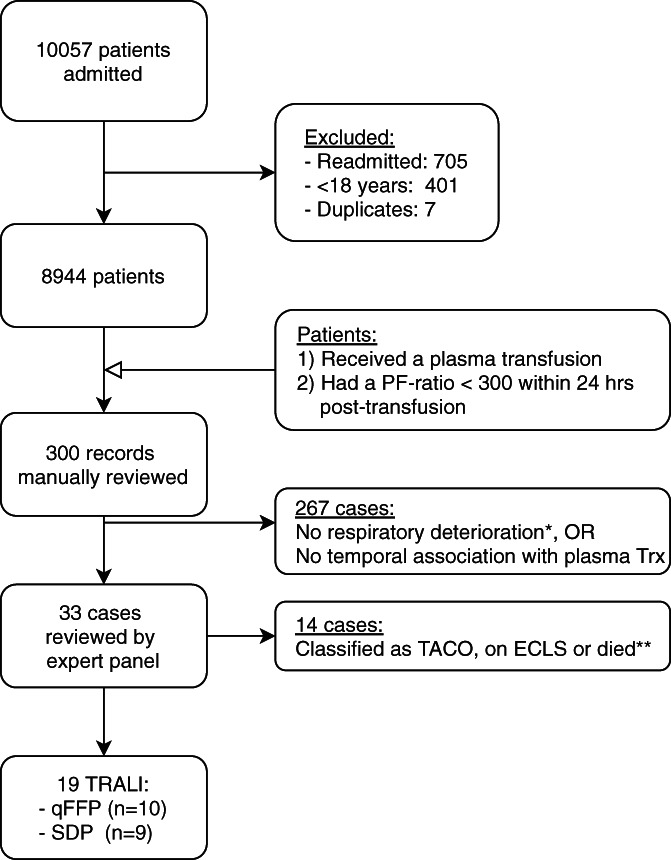
TRALI case identification and classification. Flow diagram detailing TRALI patient selection. *Respiratory deterioration defined as: increased oxygen requirements, intubation, decreased PF‐ratio, or worsening ventilatory parameters (i.e., decreased compliance, increase in FiO2, PEEP or driving pressure). **Patients who died before investigations could be performed and who had an alternate clinical explanation for deterioration were excluded. ECLS, extracorporeal life support; PF‐ratio, PaO2/FiO2‐ratio; qFFP, quarantined fresh frozen plasma; SDP, solvent‐detergent pooled plasma; TACO, transfusion‐associated circulatory overload; TRALI, transfusion‐related acute lung injury.

At least one unit of plasma was transfused to 376 patients in the qFFP period and 396 patients in the SDP group. A total of 300 patients were identified to have at least one episode in which patients received a plasma transfusion and had a P/F‐ratio <300 within 24 h pre‐ or post‐transfusion. A total of 33 cases were selected to be reviewed by the expert panel, and 19 were classified as TRALI (Figure 1).

### 3.1 Incidence and characteristics of TRALI in patients receiving plasma transfusions

The characteristics of TRALI patients are shown in Table 3. A total of 19 patients developed TRALI, with two cases involving qFFP recognized and documented in the patient's chart. A total of ten patients in the qFFP group developed TRALI after 1171 plasma transfusions (0.85% per unit transfused CI95%: 0.33%–1.4%), and nine patients in the SDP developed TRALI out of 2008 units of plasma transfused (0.45%–CI95%: 0.21%–0.79%), which was not significantly different (*p* = 0.15).

**TABLE 3:** TRALI patient characteristics

Characteristics	Period		*p*‐value
	qFFP	SDP	
TRALI patients (*n*)	10	9	
Type I	4 (40%)	2 (22%)	0.405
Type II	6 (60%)	7 (78%)	“
Age (years)	67 (65 to 73)	62 (41 to 72)	0.225
Male (*n*, %)	4 (40%)	6 (67%)	0.365
BMI	24.2 ± 3.9	25.2 ± 3.0	0.519
Apache‐II score	25 (21 to 27)	23 (21 to 32)	0.485
SAPS	51 (41 to 61)	54 (52 to 79)	0.253
Transfusion data (*n*, %)			0.930
Plasma only	2 (20%)	1 (11%)	
Addition RBC units	6 (60%)	5 (56%)	
Addition PLT units	7 (70%)	5 (56%)	
Imputability (*n*, %)			0.084
Definite	2 (20%)	0 (0%)	
Possible	2 (20%)	6 (67%)	
Probable	6 (60%)	3 (33%)	
Diagnosis (*n*, %)			0.081
Cardiac arrest	2 (20%)	1 (11%)	
Major thoracic surgery	2 (20%)	1 (11%)	
Acute abdominal aortic dissection	1 (10%)	1 (10%)	
Pneumosepsis	0 (0%)	2 (22%)	
Sepsis (other)	2 (20%)	0 (0%)	
Pancreatitis	0 (0%)	2 (22%)	
Massive hemorrhage	3 (30%)	0 (0%)	
Other	0 (0%)	3 (33%)	
Presentation			
P/F‐ratio _pre‐transfusion_	245 (205 to 306)	216 (171 to 339)	0.897
P/F‐ratio _post‐transfusion_	192 (158 to 243)	207 (164 to 365)	0.447
ΔStatic compliance (mL/cmH_2_O)^a^	−6.7 (−23 to −0.3)	−7.5 (−14 to −2.5)	1.000
Chest X‐ray (*n*, bilateral infiltrates (%))	10 (100%)	9 (100%)	1.000
ΔLeukocytes	−0.5 (−6.8 to 3.6)	3.2 (2.5 to 8.3)	0.194
ΔTemperature (°C)	−0.0 (−0.8 to 0.5)	−0.2 (−0.7 to 0.3)	0.870
Fluid balance, past 72 hrs (L)	4.1 (2.9 to 8.2)	6.0 (2.6 to 9.9)	0.807
Echocardiography (*n*), impaired (%)^†^	8 (20%)	6 (33%)	0.276
Outcomes			
Hospital LOS (days)	11 (6 to 22)	14 (3 to 27)	1.000
ICU LOS (days)	5 (4 to 10)	11 (3 to 28)	0.253
Died in ICU (*n*, %)	7 (70%)	6 (67%)	1.000

*Note*: Data presented as mean ± SD or median (IQR).

Abbreviations: LOS, length of stay; P/F‐ratio, PaO2‐FiO2‐ratio; PLT, platelet transfusion; qFFP, quarantined fresh frozen plasma; RBC, red blood cells; SAPS, simplified acute physiology score; SDP, solvent/detergent treated pooled plasma.

^a^ Static compliance: calculated as tidal volume (mL)/driving pressure (cmH_2_O).

^†^ Shown as number of patients with a recent echocardiogram performed and percentage of patients with an impaired LV‐function (moderate or worse).

Type I TRALI was present in 40% of the qFFP group and in 30% of the SDP period (*p* = 0.63), shown in Table 4. There were no significant differences between age, gender, or severity of illness, that is, APACHE‐II and SAPS scores. Patients who developed TRALI received significantly more blood products—median 22 (12–43), compared with transfused patients (without TRALI), median 3 (IQR: 1–6, p < 0.001), and plasma transfused patients: 6 (IQR: 3–12, p < 0.001).

**TABLE 4:** TRALI cases

	Case:	Age (years)	Sex	Plasma only	Imputability	TRALI classification	Diagnosis	ALI risk factors	Bilateral infiltrates	Units transfused^a^		
										Plasma	RBCs	PLTs
qFFP	Case 1	70	F	Yes	Probable	Type I	Abdominal aortic aneurysm rupture	‐	Yes	3	‐	‐
	Case 2	68	F	Yes	Possible	Type II	Sepsis, GI	Shock (non‐cardiogenic)	Yes	2	‐	‐
	Case 3	66	F	‐	Definite	Type I	Myasthenia gravis	‐	Yes	2	3	1
	Case 4	61	M	‐	Definite	Type I	Aorto‐iliac bypass graft	‐	Yes	2	1	1
	Case 5	74	M	‐	Possible	Type I	CABG with aortic valve replacement	Shock (non‐cardiogenic)	Yes	1	1	‐
	Case 6	53	F	‐	Probable	Type II	Upper GI bleeding	Shock (non‐cardiac)	Yes	3	1	2
	Case 7	66	F	‐	Probable	Type II	Surgery for pelvic trauma	Shock (non‐cardiogenic)	Yes	2	‐	1
	Case 8	76	F	‐	Probable	Type II	Pericardial tamponade	Shock (non‐cardiogenic)	Yes	5	7	1
	Case 9	65	M	‐	Probable	Type II	Cardiac arrest	Trauma	Yes	3	3	1
	Case 10	83	M	‐	Probable	Type II	CABG with aortic valve replacement	Cardiac surgery	Yes	2		2
SDP	Case 11	27	M	Yes	Probable	Type I	Acute renal failure	‐	Yes	1	‐	‐
	Case 12	81	F	‐	Probable	Type I	Cardiac arrest	‐	Yes	4	3	1
	Case 13	61	M	‐	Probable	Type II	Abdomen/pelvis trauma	Shock (non‐cardiogenic)	Yes	2	2	1
	Case 14	73	M	‐	Possible	Type II	Sepsis, GI	Shock (non‐cardiogenic)	Yes	1	‐	1
	Case 15	38	M	‐	Possible	Type II	Sepsis, GI	Pancreatitis	Yes	4	2	‐
	Case 16	41	M	‐	Possible	Type II	Thoracic aortic aneurysm dissection	Shock (non‐cardiogenic)	Yes	1	1	‐
	Case 17	66	F	‐	Possible	Type II	Pneumonia, other	Pneumonia	Yes	2	‐	2
	Case 18	62	F	‐	Possible	Type II	Pneumonia, fungal	Pneumonia	Yes	2	‐	1
	Case 19	88	M	‐	Possible	Type II	Abdominal aortic aneurysm rupture	Shock (non‐cardiogenic)	Yes	2	2	‐

^a^ Units transfused within window for developing TRALI.

Abbreviations: CABG, coronary artery bypass graft (surgery); GI, gastrointestinal.

### 3.2 Imputability of SDP as cause for TRALI

Three patients developed TRALI after receiving only plasma units. In the qFFP group, two cases, a type I and type II TRALI, scored an imputability of respectively probable and possible (Table 4). One patient receiving only SDP plasma developed TRALI after a single unit with imputability scored as probable. There were no definite cases of TRALI in the SDP group.

The patient developing TRALI after a single unit of SDP was a 27‐year‐old male admitted with a drug overdose from the emergency room to the ICU. The patient went on to develop renal failure requiring renal replacement therapy as well as liver failure. A cardiac ultrasound that was performed after admission showed normal cardiac function without valvular abnormalities. On day 4 of ICU admission, the patient received a single unit of SDP at 10 a.m. to treat coagulopathy in the absence of bleeding, in the setting of liver failure. The patient was intubated at this time, and the FiO2 acutely increased from 40% to 75% within an hour of starting the transfusion, while the P/F‐ratio worsened from 167 to 116. The heart rate increased from 127/min to 142/min, and increased vasopressor support was required to maintain mean arterial pressure above 65 mmHg. While a worsening in pulmonary function was documented, the chart did not detail a change in physical exam. However, edema was notably absent. Leukocytes were measured that morning and were 3.7**·**10^9^/L; the leukocyte count the next morning was 4.4**·**10^9^/L. The temperature increased from pre‐transfusion 37.3°C to 38.0°C within 3 h. Chest X‐rays from the morning prior to transfusion compared with the day after showed an increase in bilateral central pulmonary consolidations compared with the previous X‐ray, which did already show prominent perihilar and pulmonary vasculature with peribronchiolar cuffing. No diuretics were given during this episode. The duration of increased FiO2 was approximately 36 h, and the patient could be successfully extubated after 7 days. The patient was discharged from the ICU after 22 days and discharged home after 34 days. Pulmonary deterioration was not clinically linked by the treating physicians to a transfusion and was therefore not reported to the blood bank, and no antibody testing in either patient or transfusion product was performed.

### 3.3 Risk factors and outcomes for TRALI

ALI risk factors were not significantly different between qFFP and SDP TRALI patients (eTable 3). Hospital LOS did not differ between TRALI patients and patients receiving any transfusion. ICU LOS was longer in TRALI patients with a median of 8 days (IQR 4–18) compared with transfused patients, 3 days (IQR 2–8, *p* = 0.003), and also plasma transfused patients 3 days (IQR: 2–7, p < 0.01). Mortality in the TRALI group (70%) was significantly higher than both the transfused patient group (17.2%, p < 0.001) and the plasma transfused patients (22.0%, p < 0.001).

## 4 DISCUSSION

In this before and after implementation study of SDP, we investigated the incidence of TRALI through a retrospective chart review of all patients developing acute lung injury, temporally associated with plasma transfusions. The main findings of our study are as follows: (1) the incidence of TRALI was 1:220 units, or 0.45% (CI95%: 0.19% – 0.81%), in which SDP was transfused alone, or concomitantly with other transfusion products; (2) a single case of TRALI following the transfusion of one unit of SDP was identified with probable imputability, demonstrating another case of clinically diagnosed, SDP‐induced TRALI; and (3) the overall 70% mortality of TRALI connected to plasma transfusion in the ICU is extremely high compared with 22% in all patients receiving plasma who did not develop TRALI.

To our knowledge, only one TRALI case has been reported very recently by hemovigilance systems as a result of SDP.^13^ The absence of reported cases following SDP transfusion may have calmed clinicians' concerns of TRALI as a potential complication. However, the development of TRALI follows a two‐hit event threshold model.^1^, ^2^ In our clinical practice with critically ill patients whose underlying condition often constitutes a severe first hit, we did not expect TRALI to disappear since only a minor second hit may be required to pass the threshold and activate primed neutrophils. Our study found that the incidence of clinically diagnosed TRALI in which plasma was (concomitantly) administered did decrease from 0.85% to 0.45% per transfused unit. Nine patients developed TRALI in which SDP was concomitantly administered and cannot be ruled out as a contributor, and finally, one patient developed TRALI according to the 2019 clinical criteria after a single unit of SDP, proving again that SDP transfusion cannot be marked as completely “TRALI safe.”

TRALI is a clinical diagnosis, and positive alloantibody titers are neither required for diagnosis nor confirmation of cases.^14^ In suspected cases of TRALI, laboratory analysis confirming the presence of alloantibodies can strengthen or validate the clinician's and hemovigilance officer's belief that this is a true case. Manufacturers correctly state that tested SDP plasma products have titers of anti‐human leukocyte antigen (HLA) and anti‐human neutrophil antigen (HNA) antibodies that are below the detection limit; however, this does not exclude the presence of these antibodies, nor preclude activation of neutrophils. The findings of our study show that TRALI can still occur, and clinicians must therefore remain alert and report suspected cases. Reliance on antibody titers to confirm cases should be avoided.

Our study found that a 70% ICU mortality rate in TRALI patients is much higher than the conventional TRALI mortality rate of 5%–20%.^19^, ^20^ It should be noted that our population of critically ill patients developed TRALI on top of their underlying condition. The high mortality rates may be further explained by an indication bias of plasma transfusion where outcomes of patients receiving plasma in the context of, for example, hemorrhage or spontaneous coagulopathy are likely to be worse than patients not requiring transfusion. The subgroup of patients receiving plasma transfusion had a mortality rate of 22%, similar to a previous study.^21^ Developing TRALI on top of this appears to sharply increase the risk of death to 70%; however, this is compared with an unmatched cohort. Other ICU studies have reported similar or lower mortality rates of 41% and 67%^6^, ^7^ in medical ICU patients developing TRALI.

Our study does have a number of limitations. First, the incidence of TRALI reported is not the true incidence of TRALI in the ICU. Only patients receiving plasma were reviewed, thereby excluding TRALI cases due to other blood products. Second, this was a database study where imprecise registration of the transfusion start and end times could have led us to underestimate the number of cases as TRALI. The diagnosis of TRALI was ruled out beyond the six‐hour post‐transfusion window. On the other hand, a retrospective study of cases has the potential for confirmation bias and overestimation of the number of TRALI cases. Also, there is a risk of misdiagnosing TRALI as TACO, which can be difficult to differentiate retrospectively. We minimized this by utilizing an expert panel to adjudicate cases, which was blinded to the year the case occurred and to which plasma product was transfused. Furthermore, due to the retrospective nature of the study, the diagnosis of TRALI was made based on clinical criteria, antibody measurements in the suspected products were not performed, and a causal relationship cannot be determined. Moreover, donor antibody screening is not feasible when implicated products contain between 300 and 500 donors. Finally, in cases where more than one blood product was transfused within the 6‐h TRALI window, it was impossible to discern a single unit as the culprit. Whether these cases were due to SDP or whether SDP was an innocent bystander remains unclear.

Based on our findings, we advocate that clinicians remain vigilant when transfusing SDP. Cases of TRALI can still occur, and early recognition and supportive care for these patients are critical. Unrecognized cases of TRALI will certainly not help combat the extremely high mortality seen. Furthermore, additional investigation is needed to understand whether diluted HLA or HNA antibodies are still able to induce TRALI in the presence of a first hit or that other substances in SDP are causative. Sources labeling SDP as safe, “TRALI free”, or TRALI as abolished in the setting of plasma transfusion may lull clinicians into a false sense of security, which may delay recognition and prompt stabilization of these patients. Suspicion should remain high and laboratory work‐ups should be performed according to hospital protocol.

## 5 CONCLUSION

Implementation of SDP has decreased the incidence of TRALI; however, clinically diagnosed cases of TRALI still occur following the transfusion of SDP. Clinicians should remain vigilant and continue to report cases. TRALI cases involving plasma transfusion in the ICU are associated with very high mortality.

## FUNDING INFORMATION

The research was funded by a personal grant from the Dutch Research Council (NWO) awarded to Prof. Dr. Alexander P.J. Vlaar. VIDI grant (number: 09150172010047). In this investigator‐initiated study, the funding bodies were in no way involved in the study design, collection, analysis, and interpretation of data, nor in writing the manuscript.

## CONFLICT OF INTEREST

The authors have disclosed no conflicts of interest.

## Supporting information


**eTable 1.** Imputability scoring.
**eTable 2.** Transfusion products.
**eTable 3.** TRALI patient characteristics.
