# Supplementary material for: Incidence of transfusion‐related acute lung injury temporally associated with solvent/detergent plasma use in the ICU: A retrospective before and after implementation study
Source: Transfusion. 2022 Aug 2;62(9):1752–62. doi: 10.1111/trf.17049 (PMC9544437; doi:10.1111/trf.17049)
Supplement: Supplementary file 1 — eTable 1. Imputability scoring. eTable 2. Transfusion products. eTable 3. TRALI patient characteristics. [file TRF-62-1752-s001.doc]

**eTable 1.** Imputability scoring

| **Imputability** | **Definition** |
| --- | --- |
| Definite | when there is conclusive evidence beyond reasonable doubt that the adverse event can be attributed to the transfusion |
| Probable | when the evidence is clearly in favor of attributing the adverse event to the transfusion |
| Possible | when the evidence is indeterminate for attributing the adverse event to the transfusion or an alternate cause |
| Dou**btful** | when the evidence is clearly in favor of attributing the adverse event to causes other than the transfusion |
| E**xcluded** | when there is conclusive evidence beyond reasonable doubt that the adverse event can be attributed to causes other than the transfusion |

Adapted from: International Society of Blood Transfusion – Working Party on Haemovigilance, 2013. [15]

**eTable 2.** Transfusion products

| **Characteristics** | **Volume** | **Fabrication process** | **Contains plasma** |
| --- | --- | --- | --- |
| Red blood cells (RBCs) | 280 mL | 1. Whole blood donation stored in a citrate-phosphate-dextrose-solution 2. Centrifuged and sorted into i.a. an erythrocyte component  3. Saline-adenine-glucose-mannitol storage solution is added  4. Product is leucoreduced | Contains < 20% plasma |
| Platelet concentrate (PC) | 280 mL | 1. Buffy-coat from 5 whole blood donations are pooled  2. Platelet additive solution C is added  3. Buffy-coat pools are centrifuged and leucoreduced | ± 35% of suspension volume is plasma |
| Plasma: |  |  |  |
| - qFFP | 280 mL | 1. A single donor unit of apheresis plasma is collected and frozen | Contains 100% plasma |
| - - SDP | 280 mL | 1. A single donor unit of apheresis plasma is collected and frozen  2. Donations are pooled, and residual cells are filtered  3. Solvent-detergent treatment for virus inactivation  4. Prion reduction  5. Sterile filtration  4. Aliquoting and refreezing |

Blood products in the Netherlands are fabricated from unpaid volunteer donations to the Dutch national blood bank (*Sanquin, Amsterdam – The Netherlands*). Sanquin also produces most blood products following national guidelines. SDP made from unpaid, male only volunteer donated plasma and processed. Abbreviations: *qFFP:* quarantine single unit fresh frozen plasma; *SDP:* solvent/detergent treated pooled plasma.

**eTable 3.** TRALI patient characteristics

| **Characteristics** | **Period** | | **p-value:** |
| --- | --- | --- | --- |
|  | **qFFP** | **SDP** |  |
| TRALI patients, n | 10 | 9 |  |
| Patients transfused |  |  |  |
| - Units per transfused patient | 23 (12 – 40) | 22 (11 – 44) | 0.870 |
| - RBC units | 13 (8 – 18) | 11 (7 – 17) | 0.742 |
| - Plasma units per patient | 7 (2 – 16) | 4 (4 – 9) | 0.741 |
| - PLT units | 5 (3 – 5) | 6 (3 – 7) | 0.709 |
| Products transfused (n) | 284 | 229 | 0.015 |
| - RBCs | 141 | 102 | 0.012 |
| - PLTs | 44 | 48 | 0.677 |
| - Plasma | 99 | 79 | 0.134 |
| - Plasma units (vol. corrected)* | 99 | 51 | <0.001 |
| Type of admission, n (%) |  |  | 0.234 |
| - Medical | 3 (30%) | 6 (67%) |  |
| - Emergency surgery | 4 (40%) | 3 (33%) |  |
| - Planned surgery | 3 (30%) | 0 (0%) |  |
| Comorbidities, n (%) |  |  |  |
| - Chronic renal disease | 0 (0%) | 0 (0%) | - |
| - COPD | 0 (0%) | 0 (0%) | - |
| - Hematological malignancy | 0 (0%) | 2 (22%) | 0.207 |
| - Immunological insufficiency | 0 (0%) | 2 (22%) | 0.213 |
| - Diabetes | 0 (0%) | 1 (11%) | 0.476 |
| - History of heart failure | 1 (10%) | 0 (0%) | 1.000 |
| - Cirrhosis | 1 (10%) | 0 (0%) | 1.000 |
| Risk factors, n (%) |  |  |  |
| - Direct |  |  |  |
| - - Pneumonia | 0 (0%) | 2 (22%) | 0.203 |
| - - Aspiration | 0 (0%) | 0 (0%) | - |
| - - Inhalation: smoke/drowning | 0 (0%) | 0 (0%) | - |
| - Indirect |  |  |  |
| - - Sepsis | 1 (10%) | 2 (22%) | 0.584 |
| - - Trauma | 1 (10%) | 1 (11%) | 1.000 |
| - - Pancreatitis | 0 (0%) | 1 (11%) | 0.476 |
| - - Drug overdose | 0 (0%) | 1 (12%) | 0.476 |
| - Other |  |  |  |
| - - Cardiac surgery | 1 (10%) | 0 (0%) | 1.000 |
| - - Cardiac Arrest | 1 (10%) | 1 (11%) | 1.000 |

Data presented as mean ± SD or median (IQR); Abbreviations: *RBC:* red blood cells; *PLT*: platelet transfusion; *qFFP:* quarantine single unit fresh frozen plasma; *SDP:* solvent/detergent treated pooled plasma; *COPD:* chronic obstructive pulmonary disease.
